# Survival of glioblastoma in relation to tumor location: a statistical tumor atlas of a population-based cohort

**DOI:** 10.1007/s00701-021-04802-6

**Published:** 2021-03-20

**Authors:** Even Hovig Fyllingen, Lars Eirik Bø, Ingerid Reinertsen, Asgeir Store Jakola, Lisa Millgård Sagberg, Erik Magnus Berntsen, Øyvind Salvesen, Ole Solheim

**Affiliations:** 1 Department of Radiology and Nuclear Medicine, St. Olavs Hospital, Trondheim University Hospital, Prinsesse Kristinas Gate 1, 7006 Trondheim, Norway; 2 Department of Neuromedicine and Movement Science, Faculty of Medicine and Health Sciences, Norwegian University of Science and Technology (NTNU), Trondheim, Norway; 3 Department of Health Research, SINTEF Digital, Trondheim, Norway; 4 Department of Neurosurgery, Sahlgrenska University Hospital, Gothenburg, Sweden; 5 Institute of Neuroscience and Physiology, Department of Clinical Neuroscience, Sahlgrenska Academy, University of Gothenburg, Gothenburg, Sweden; 6 Department of Neurosurgery, St. Olavs Hospital, Trondheim University Hospital, Trondheim, Norway; 7 Department of Public Health and Nursing, Faculty of Medicine and Health Sciences, Norwegian University of Science and Technology (NTNU), Trondheim, Norway; 8 Department of Circulation and Medical imaging, Faculty of Medicine and Health Sciences, Norwegian University of Science and Technology (NTNU), Trondheim, Norway

**Keywords:** General population, Glioblastoma, MRI, Neurosurgery, Survival, Tumor atlas

## Abstract

**Purpose:**

Previous studies on the effect of tumor location on overall survival in glioblastoma have found conflicting results. Based on statistical maps, we sought to explore the effect of tumor location on overall survival in a population-based cohort of patients with glioblastoma and *IDH* wild-type astrocytoma WHO grade II–III with radiological necrosis.

**Methods:**

Patients were divided into three groups based on overall survival: < 6 months, 6–24 months, and > 24 months. Statistical maps exploring differences in tumor location between these three groups were calculated from pre-treatment magnetic resonance imaging scans. Based on the results, multivariable Cox regression analyses were performed to explore the possible independent effect of centrally located tumors compared to known prognostic factors by use of distance from center of the third ventricle to contrast-enhancing tumor border in centimeters as a continuous variable.

**Results:**

A total of 215 patients were included in the statistical maps. Central tumor location (corpus callosum, basal ganglia) was associated with overall survival < 6 months. There was also a reduced overall survival in patients with tumors in the left temporal lobe pole. Tumors in the dorsomedial right temporal lobe and the white matter region involving the left anterior paracentral gyrus/dorsal supplementary motor area/medial precentral gyrus were associated with overall survival > 24 months. Increased distance from center of the third ventricle to contrast-enhancing tumor border was a positive prognostic factor for survival in elderly patients, but less so in younger patients.

**Conclusions:**

Central tumor location was associated with worse prognosis. Distance from center of the third ventricle to contrast-enhancing tumor border may be a pragmatic prognostic factor in elderly patients.

**Supplementary Information:**

The online version contains supplementary material available at 10.1007/s00701-021-04802-6.

## Introduction

Glioblastomas grow into surrounding brain tissue in a diffusely infiltrating pattern. Prognosis is poor with 5-year survival around 5% [30], but with large inter-individual differences. Accurate prognostication at the individual level is difficult, but desirable to avoid both over-treatment and under-treatment. In addition to functional level and age, tumor size and location is usually a major part of the clinical decision-making process prior to surgery. Surgical extent of resection is positively linked to survival, though surgery seemingly needs to be extensive to have a meaningful independent impact on survival [8].

Surgical operability and “safe” extent of resection in a given case is highly subjective and depends much on the anatomical location [34, 47]. Although eloquence of tumor location may be graded according to the Sawaya classification [35], there is no common understanding on how to assess risks and which regions to carefully avoid, resulting in high variability in treatment strategies [4, 28]. It has previously been reported that tumor location in glioblastoma is associated with several important prognostic factors including age [10], extent of resection [41], and molecular markers such as isocitrate dehydrogenase 1 (*IDH1*) mutation and possibly O^6^-methylguanine DNA methyltransferase (*MGMT*) promoter methylation [10, 23, 45]. However, studies exploring the importance of tumor location in relation to overall survival (OS) have found in part conflicting results [10, 24, 32].

Methods for three-dimensional (3D) volumetric segmentation of brain tumors based on magnetic resonance imaging (MRI) scans are becoming increasingly available [26]. These methods provide more accurate and precise measurements of tumor volume and location, and allow for construction of statistical tumor maps, i.e., voxel-based maps of tumors registered to a standardized brain model. Such maps enable exploration of effects on clinical endpoints, e.g., extent of resection, survival, morbidity, and quality of life (QoL) [10, 19, 24, 33].

The aim of this population-based cohort study was to explore the impact of tumor location on OS based on statistical tumor maps constructed by segmentation of preoperative 3D MRI scans in patients with histopathologically verified WHO grade IV glioma (glioblastoma) and histopathologically verified *IDH1* wild-type WHO grade II–III astrocytoma with radiological necrosis.

## Material and methods

Consecutive glioma patients with age ≥ 18 years eligible for tumor resection or biopsy only between January 2007 and December 2016 were identified from the surgery database at the Department of Neurosurgery, St. Olavs Hospital, Trondheim, Norway. This department exclusively serves a defined geographical catchment region. Preoperative contrast-enhanced (CE) 3D T1-weighted cerebral MRI scans are routinely acquired in glioma patients < 72 h before surgery. A tissue diagnosis is advocated in patients with suspected glioma, ensuring a population-based case selection of patients with histopathologically confirmed tumors. Patients with histopathologically verified diffuse glioma grade IV, and patients with histopathologically verified *IDH1* wild-type astrocytoma grade II–III with CE components and necrotic cores on MRI scans were eligible for inclusion. *IDH1* status in grade II–III tumors was assessed by IDH1 R132H immunohistochemistry. The latter patient group was included as there is a risk of non-representative biopsies in histopathological tumor verification, and surgery in these patients was planned and performed as in glioblastoma patients. Furthermore, *IDH* wild-type astrocytoma grade II–III has expected survival on par with glioblastoma [3]. These patients are treated as grade IV glioma at our institution. *IDH* mutation status was not available for approximately half the population with histopathologically verified glioblastoma and therefore not included as a variable in the analyses. Patients without CE 3D T1 MRI scans prior to surgery, patients with non-CE/poor and diffuse CE tumors, patients who had undergone previous tumor resection, and patients with multifocal tumors were excluded. Surgeries were performed under general anesthesia, and a neuronavigation system based on preoperative MRI scans and intraoperative 3D ultrasound was routinely used [46].

The Karnofsky Performance Status (KPS) score was recorded prospectively by the operating surgeon in the majority of patients. In cases with missing data for KPS (*n* = 57, 27%), a retrospective KPS dichotomized to < 70 (dependent) or ≥ 70 (functionally independent) was obtained based on preoperative functional status description in the admission papers.

Patients were postoperatively referred to the oncology department for evaluation of radiochemotherapy in accordance with the Stupp protocol [43]. Patients who started both radiotherapy and temozolomide treatments within 3 months after primary surgery were categorized as receiving radiochemotherapy. Patients who started but did not complete radiochemotherapy were categorized as receiving radiochemotherapy on an intention-to-treat basis. Patients only receiving either radiotherapy or chemotherapy were categorized as not receiving radiochemotherapy. Any adjuvant chemotherapy treatment received beyond the standard postoperative cycles of temozolomide after surgery was not included in the study variables. Patients were followed until death or censored 31.12.2018, whichever came first. Survival in days was calculated from date of surgery/biopsy to date of death/censored.

Preoperative tumor volume was defined as the CE tumor border plus the necrotic core on T1 MRI scans. Tumor segmentation was performed on preoperative 1.5T or 3T CE 3D T1 MRI scans using two different software packages: BrainVoyager^TM^ QX 1.2 [15] and 3D Slicer version 4.3.1.-4.10.2 [7]. We have previously demonstrated high agreement between these software packages [14]. The workflow has been described in detail in other publications [7, 14, 40]. Residual tumor volume (RTV) was defined as CE components only in resected patients, in accordance with previous studies [8, 41]. In cases with biopsy only, RTV was set equal to preoperative tumor volume (including necrotic core). Segmentations were performed by two of the authors (E.H.F, L.M.S), or trained medical students (A.L.S, J.S). All segmentations were verified by a resident radiologist (E.H.F), a neurosurgeon (O.S), or a neuroradiologist (E.M.B). Residual tumor volumes were segmented by E.H.F or a resident neurosurgeon (P.M) and subsequently verified by E.H.F. In 10 cases who underwent surgery, there were no postoperative 3D MRI scans. These cases were included in the survival maps but excluded from regression analyses that included RTV.

Segmented images were registered to the standardized frame of reference known as the Montreal Neurological Institute (MNI) space, defined by the ICBM-152 brain template [13]. Registration to MNI space is described in a previous publication [33]. The registered tumor segmentations were combined into a tumor distribution map showing the number of patients with tumor in each voxel.

Statistical maps were created to explore OS in relation to tumor location. The procedure was partly based on the procedure described by de Witt Hamer et al. [6]. Patients were divided into groups with short OS (< 6 months), medium OS (6 months to 24 months), and relatively long OS (> 24 months). Tumor odds maps were created for each OS group by dividing the number of patients with tumor in each voxel by the number of patients without tumor in the same voxel, indicating the odds of a patient in a given group having a tumor in a given voxel. To determine if the presence of a tumor in an area of the brain was associated with OS of the patient, the three tumor odds maps were compared in pairs using a voxel-wise approach. First, the log odds ratio of each voxel was computed by calculating the natural logarithm of the ratio between the odds from the two maps, creating a log odds ratio map. For any voxel in which no patients in an OS group had a tumor, the number of cases for that group in that voxel was set to 0.000001 for computational purposes. To determine the significance of the difference between the two odds, a 2×2 contingency table was created for each voxel, with the OS group as one variable and the presence or absence of tumor as the other. Fisher’s exact test was used to test the significance of the association between the two variables, resulting in three *p*-value maps (OS 6–24 months vs. < 6 months, OS > 24 months vs. 6–24 months, and OS > 24 months vs. < 6 months). The odds of having a tumor in a voxel are related to the odds of having a tumor in the neighboring voxels. This spatial dependency was accounted for by randomly permuting the list of OS groups so that each patient (and the corresponding tumor) was assigned a new, random OS group, and then calculating a new *p*-value for each voxel based on this permutation. This was repeated 2000 times resulting in a null-distribution of 2000 random *p*-values per voxel. The adjusted *p*-values were then calculated as the proportion of *p*-values in the null-distribution that are smaller than the original *p*-value. The significance level of adjusted *p*-values was set to ≤ 0.01 due to multiple testing between groups. A statistical map was created where all voxels with a statistically significant adjusted *p*-value was set to one and the rest were set to zero. This map was then used to mask the log odds ratio map so that only voxels with adjusted *p*-value ≤ 0.01 were visualized.

Based on the findings of the maps, the shortest distance from the center of the third ventricle to contrast-enhancing tumor border (TVTB) in centimeters emerged as a potential prognostic factor and was calculated using the Hammersmith atlas [17].

Cox proportional hazard regression was performed to ascertain the effect of covariates on OS. Eligible covariates for the multivariable Cox regression were age, sex, preoperative KPS (< 70 or ≥ 70), biopsy only (yes/no), postoperative radiochemotherapy (yes/no), preoperative tumor volume in milliliters, RTV in milliliters, and TVTB in centimeters. Multiple possible interaction terms were explored with significance level for inclusion in the multivariable regression model set to *p* ≤ 0.01 due to multiple testing. Likewise, binomial logistic regression models were performed to explore predictive factors for adjuvant radiochemotherapy and biopsy only. Ordered logistic regression models were performed to explore predictors of RTV. For this analysis only, RTV was grouped into interval groups of 5 mL in accordance with a previous study [8]. Patients with biopsy only were not included in the ordered logistic regression models exploring predictors of RTV. Covariates with *p ≤* 0.1 in the univariable analyses were included in the multivariable models for all regression analyses. Details of possible interaction terms and statistical specifications for the regression models are provided in Online Resource 1.

## Results

**Fig. 1:**
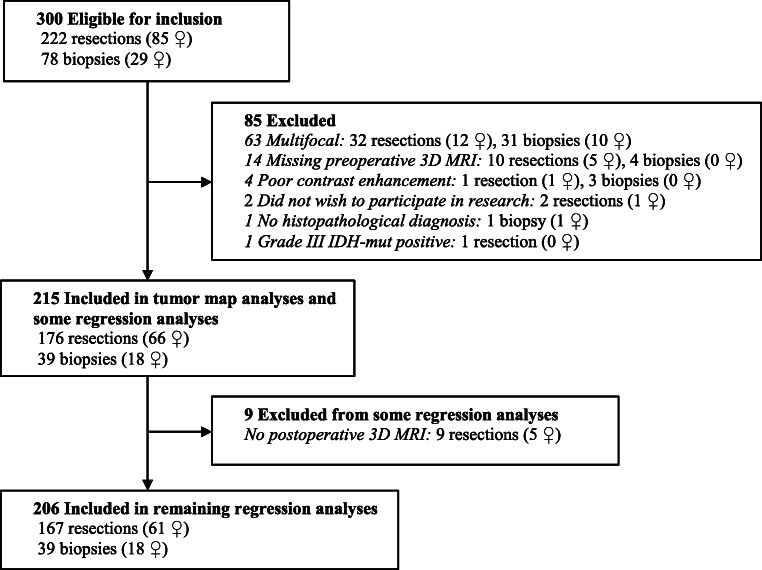
Flow chart of the inclusion process

A total of 215 patients (84 women) were included in the tumor map analyses. One patient (man) had *IDH1* wild-type astrocytoma grade II with radiological necrosis, while 15 patients (10 women) had *IDH1* wild-type astrocytoma grade III with radiological necrosis. The remaining 199 patients had histopathologically verified glioblastoma. A total of 206 patients were included in the Cox regression analyses. All nine patients alive at time of censoring had survival > 24 months. A flow chart of the inclusion process is presented in Fig. 1.

Descriptive statistics are presented in Table 1. Tumor resection was performed in 176 patients (82%). The majority of patients were functionally independent with KPS ≥ 70 prior to surgery (*n* = 150, 70%) and received adjuvant radiochemotherapy (*n* = 165, 77%). Median survival in the entire population was 374 days.

**Table 1:** Descriptive statistics of the patient population (*N* = 215)

Sex, *n* (%)	
Female	84 (39.1)
Male	131 (60.9)
Age in years, median (range)	65 (24 to 89)
Preoperative KPS, *n* (%)	
≥ 70	150 (69.8)
Resection, *n* (%)	
Yes	176 (81.9)
Preoperative tumor volume, median (range)	34.3 (0.4 to 243.5)
Residual tumor volume in mL^a^ (*n* = 206), median (range)	
Resection (*n* = 167)	1.63 (0 to 68.1)
Biopsy (*n* = 39)	35.99 (0.97 to 144.38)
Residual tumor volume grouped^a^ (*n* = 206), *n* (%)	
0–5 mL	120 (58.3)
5.1–10 mL	26 (12.6)
10.1–15 mL	15 (7.3)
15.1–20 mL	7 (3.4)
> 20 mL	38 (18.4)
Radiochemotherapy, *n* (%)	
Yes	165 (76.7)
Survival in days, median (range)	374 (8 to 2924)
Survival grouped, *n* (%)	
< 6 months	52 (24.2)
6–24 months	122 (56.7)
> 24 months	41 (19.1)

^a^Residual tumor volume was calculated as contrast enhancing components only in patients treated with resection. Residual tumor volume was set equal to preoperative tumor volume (including necrotic parts) in patients treated with biopsy only

**Fig. 2:**
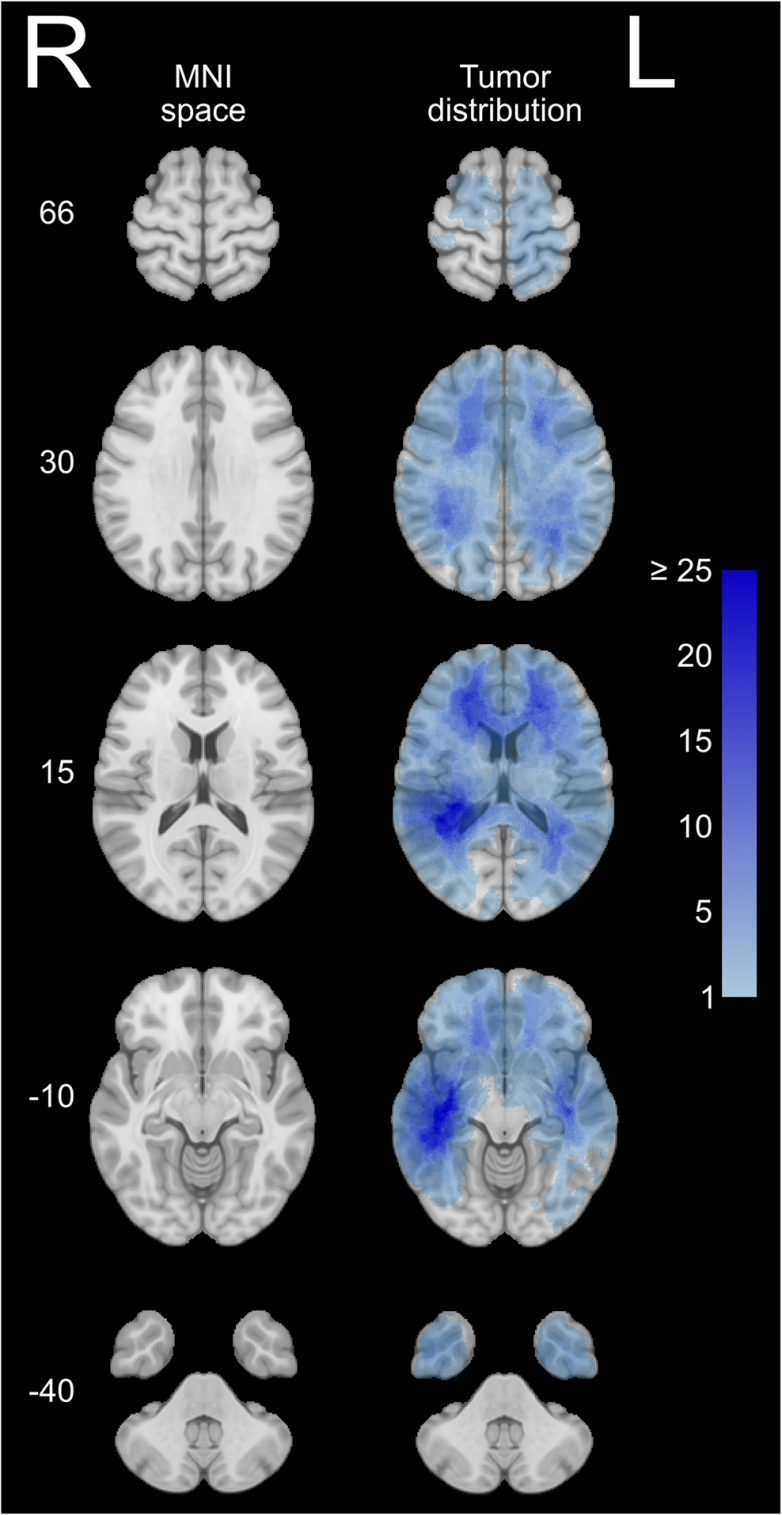
Left: overview of the Montreal Neurological Institute (MNI) space. Right: distribution map of all patients (resection + biopsy, *N* = 215) with increasing number of cases in each voxel from light blue to dark blue

**Fig. 3:**
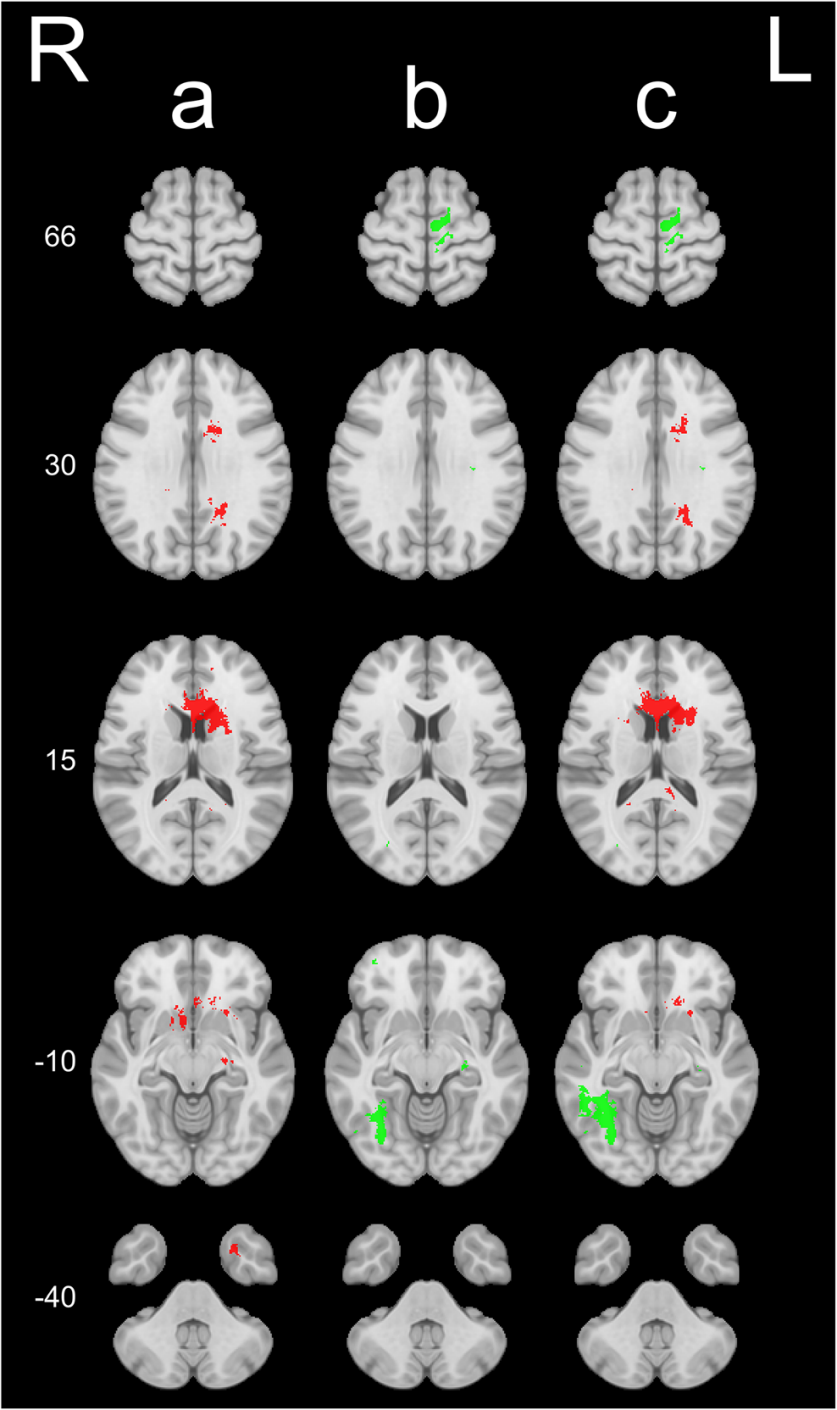
Log odds ratio maps for voxels with *p* ≤ 0.01 showing differences in overall survival (OS) based on tumor location (resection + biopsy) between (a) OS 6–24 months vs. OS < 6 months (*n* = 174), (b) OS > 24 months vs. OS 6–24 months (*n* = 163), and (c) OS > 24 months vs. OS < 6 months (*n* = 93). Green voxels have positive log odds ratios, which imply higher tumor odds in the first of the two groups, and thus indicate higher OS for patients with tumors in these areas. Red voxels have negative log odds ratios, which imply lower tumor odds in the first of the two groups, and thus indicate lower OS for patients with tumors in these areas

Overview of the tumor distribution of the entire cohort is presented in Fig. 2. Maps with statistically significant differences in tumor location between the three groups (OS < 6 months, OS 6–24 months and OS > 24 months) are presented in Fig. 3. Tumor location in central structures including the anterior half and splenium of corpus callosum and basal ganglia was associated with OS < 6 months. Furthermore, tumor location in the left temporal lobe pole was association with OS < 6 months. Contrarily, tumor location in the dorsomedial right temporal lobe including ventral parts of the occipital lobe, and white matter in the region of the left anterior paracentral gyrus/dorsal supplementary motor area/medial precentral gyrus, and small regions of the left centrum semiovale, medial left temporal lobe and dorsolateral left temporal lobe was associated with OS > 24 months.

As the maps showed reduced OS in centrally located tumors, we hypothesized that TVTB could serve as a pragmatic prognostic factor for survival. The results from the post-hoc Cox regression are presented in Table 2. There was a statistically significant interaction between age and TVTB (age × TVTB), and this interaction term was included in the multivariable model. In the multivariable model, increasing age and RTV were negative predictors for OS with hazard ratio (HR) 1.082 (95% CI 1.040 to 1.125) and 1.016 (95% CI 1.006 to 1.027), respectively. Preoperative higher KPS and adjuvant radiochemotherapy were positive predictors for OS with HR 0.546 (95% CI 0.396 to 0.775) and 0.384 (95% CI 0.258 to 0.573), respectively. Due to the interaction between age and TVTB, the HR for TVTB decreases with increasing age in the multivariable analyses (HR for TVTB 2.406, 95% CI 1.206 to 4.802 and HR for interaction term [age × TVTB] 0.983, 95% CI 0.972 to 0.994).

**Table 2:** Hazard ratio (HR) of patient characteristics, tumor characteristics, adjuvant treatment, and mortality in both resected in biopsied tumors (*N =* 206)

	Univariable		Multivariable	
	HR (95% CI)	*p*	HR (95% CI)	*p*
Age (per year)	1.035 (1.020–1.050)	< 0.001	1.082 (1.040–1.125)	< 0.001
Sex (reference female)				
Male	1.230 (0.918–1.649)	0.166	-	-
Preoperative KPS (reference ≤ 70)				
≥ 70	0.445 (0.327–0.606)	< 0.001	0.546 (0.385–0.775)	0.001
Preoperative tumor volume (per mL)	1.008 (1.004–1.012)	< 0.001	0.999 (0.992–1.005)	0.672
TVTB^a^ (per cm)	0.770 (0.696–0.852)	< 0.001	2.406 (1.206–4.802)	0.013
Biopsy only (reference no)				
Yes	3.208 (2.222–4.633)	< 0.001	1.242 (0.675–2.285)	0.485
Residual tumor volume (per mL)	1.025 (1.020–1.031)	< 0.001	1.016 (1.006–1.027)	0.003
Adjuvant radiochemotherapy (reference no)				
Yes	0.257 (0.180–0.368)	< 0.001	0.384 (0.258–0.573)	< 0.001
Age × TVTB^b^	-	-	0.983 (0.972–0.994)	0.002

^a^TVTB—shortest distance from center of 3. Ventricle to preoperative contrast-enhancing tumor border

^b^Interaction term between age and TVTB

Results from the ordered and binomial logistic regression analyses are presented in Online Resources 2–4. Preoperative KPS, preoperative tumor volume, and TVTB were predictors of RTV with OR 0.439 (95% CI 0.195 to 0.987), 1.015 (95% CI 1.002 to 1.028), and 0.527 (95% CI 0.358 to 0.774), respectively. Furthermore, increasing age and TVTB were predictors for treatment with biopsy only with OR 1.149 (95% CI 1.089 to 1.212) and 0.508 (95% CI 0.349 to 0.743), respectively. Lastly, age, KPS, and TVTB were predictors for treatment with adjuvant radiochemotherapy with OR 0.879 (95% CI 0.830 to 0.930), 3.811 (95% CI 1.660 to 8.749), and 1.531 (95% CI 1.002 to 2.339), respectively.

## Discussion

This is the first population-based study to explore OS of glioblastoma patients based on a statistical tumor atlas. Tumors affecting central brain structures (corpus callosum, basal ganglia) and left temporal lobe pole were associated with survival < 6 months. Tumors involving the dorsomedial right temporal lobe, and the white matter region involving the left anterior paracentral gyrus/dorsal supplementary motor area/medial precentral gyrus were associated with survival > 24 months. Our results indicate that TVTB as a proxy for centrally located tumors is a prognostic factor for OS, but that this effect may be age-dependent with increasing effect of TVTB with increasing age.

Preoperative prognostication in glioblastoma is difficult and several of the established prognostic markers may perhaps be associated with treatment nihilism, and their clinical impact may therefore in part represent self-fulfilling prophesies. The impact of central tumor location may also be a marker of treatment nihilism. Still, centrally located cancers have short route to white matter tracts, and both tumor progression along such tracts [11] and adverse effects from surgery may result in reduced OS.

As glioblastoma surgery has a relatively high risk of complications, sequelae, and loss of quality of life in a patient population with poor long-term prognosis [16, 20], identifying patients who are unlikely to benefit from surgery is imperative. Previous studies on survival in relation to tumor location have reported reduced survival with periventricular involvement and a possible survival difference between left- and right-sided tumors [1, 10, 24, 32]. One study found higher number of patients with survival < 12 months in the right temporal lobe and a higher number of patients with survival > 36 months in the left temporal lobe. There were smaller regions in the medial and posterior left temporal lobe associated with OS > 24 months in our study, but tumors in the left temporal lobe pole were associated with OS < 6 months. Furthermore, tumors in the dorsomedial right temporal lobe were associated with OS > 24 months. Another study found reduced survival (< 11 months) in patients with tumor involving a small region of the occipitotemporal periventricular white matter, but no differences between right- and left-sided tumors [24]. A third study of *IDH* wild-type glioblastoma found reduced OS (< 14.4 months) with involvement of several structures, including periventricular white matter/corpus callosum and deep structures (basal ganglia, thalami) [32]. However, these studies may have limited generalizability in that results are based on data from referral centers, with the risk of selection bias. Our maps showed an association between OS > 24 months and white matter tumors in the region of the left anterior paracentral gyrus/supplementary motor area/medial precentral gyrus. This may reflect an earlier diagnosis due to detectable problems with fine motor deficits of the dominant leg or hand. Compared to previous studies using statistical maps, we used a different set of cut-offs for short- and long-term survivors (< 6 months and > 24 months, respectively) [10, 24]. With median OS in unselected glioblastoma patients of 10 months [21], we argue that identifying patients with a clearly poor prognosis (< 6 months) provides more useful information to neurosurgeons for when surgery is less likely to be beneficial. Few patients live > 36 months, and > 24 months was therefore assessed to be a good outcome.

Differences in gene expressions between hemispheres have been reported [44], with the potential of affecting survival through location-dependent differences in biomarkers associated with OS. Our regression analyses do not include status of *IDH* mutation, *MGMT* promoter methylation, or *TERT* mutation, which are associated with survival [9, 37, 42]. *IDH1*-mut tumors with a favorable prognosis have been reported to be more common in the frontal lobes in proximity to the anterior ventricles and involving the anterior corpus callosum, and possibly also in the left insular region [23, 45]. These results do not clearly correspond to our findings, with maps indicating reduced OS in tumors involving the corpus callosum. The same studies did not find *IDH1* wild-type tumors to have a location predilection [23, 45]. However, only about 9% of glioblastomas have *IDH*-mut [29]. Furthermore, *IDH*-mut are hallmarks of secondary glioblastomas, and a large portion of *IDH*-mut tumors are diagnosed and undergo first-time surgery as grade II/III gliomas. In a previous study from our institution on 106 glioblastoma patients with a partly overlapping patient population, only two cases had *IDH1* mutations [39]. It is likely that our study population includes only a small number of *IDH*-mut glioblastomas, and all included grade II and III tumors were *IDH1* wild-type. Previous studies on *MGMT* status and tumor location are conflicting [10, 45]. However, the largest of these studies found that *MGMT* unmethylated tumors with an unfavorable prognosis were more common in the right hemisphere [10]. This does not clearly correlate with our findings of an association between tumors in the dorsolateral right temporal lobe and survival > 24 months. *TERT* mutation has not been associated with tumor location [37, 45]. *H3F3A* K27M mutation is associated with midline gliomas [27], but OS in K27M mutated compared to K27M wild-type high-grade gliomas in adults is similar [27, 36], and is therefore less likely to explain location-dependent differences in OS. Although previous studies on location differences in known prognostic mutations do not clearly align with our findings, we cannot exclude that location-dependent mutations in part may explain the survival differences in our maps. However, molecular markers are not available prior to first-time surgery and are therefore of limited relevance in surgical decision-making.

Surgically acquired neurological deficits are associated with reduced OS [25] and increased risk of not receiving adjuvant radiochemotherapy [16]. Previous studies exploring the risks of surgery involving central brain structures are conflicting [5, 12, 35]. Prognostic factors for OS such as KPS, preoperative neurological function, and RTV are likely to be correlated with tumor location due to proximity to eloquent structures and increased technical surgical difficulties in central regions. The individual effect size of these factors was not possible to explore in our map-based approach, as such analyses would require a very large dataset. However, our post hoc regression analyses showed that increasing TVTB was a prognostic factor for OS, indicating an independent effect of central tumor location. Interestingly, the association between TVTB and OS was dependent on age (identified as a statistically significant interaction between age and TVTB), with increasing effect of tumor proximity to central structures with increasing age. For example, the HR of TVTB in a 60-year-old patient in our study was 0.86, while it was 0.61 in an 80-year-old patient. The brain undergoes age-dependent atrophy with decline in function [31], and elderly patients have reduced rehabilitation potential compared to younger patients after ischemic stroke [22]. *IDH1* wild-type tumors are also more common in elderly patients, partly explaining the age-dependent prognosis of glioblastoma survival [18]. *MGMT* promoter methylation is less clearly associated with age [2, 38]. Thus, the age-dependent effect of TVTB in our study may result from reduced potential of rehabilitation after surgery, age-dependent nihilism of treatment at relapse, and possible age-dependent differences in tumor biology.

Lower TVTB was a predictor for biopsy only, increased RTV, and not receiving adjuvant radiochemotherapy. This indicates that neurosurgeons at our institution pursue a less aggressive surgical approach with more centrally located tumors, and that TVTB may also influence the neuro-oncologists’ assessment of adjuvant treatment. Due to the known association between OS and both RTV and radiochemotherapy, a more aggressive surgical and oncological approach could potentially increase OS also in patients with centrally located tumors, though risk of neurological sequelae would also likely increase. This illustrates the difficulties in weighing the pros and cons of treatment in the individual patient. However, the analyses did not include assessment of cognitive function, which may correlate with central tumor location and influence the decision-making process of both neurosurgeons and neuro-oncologists.

This statistical atlas is constructed based on a total of 215 glioblastoma cases. As such, there are areas of the brain with no or only a small number of tumors in each voxel. For example, there were only 41 patients with survival > 24 months resulting in a low tumor count in each voxel. Some differences between maps in Fig. 3 may therefore be a result of lower power in the OS > 24 months vs. OS < 6 months map. Our results based on the statistical atlas should therefore be repeated in larger data sets. By use of segmentation, there is a risk of errors of the exact contrast-enhancing borders of the tumors, and registration to the standardized MNI space is associated with small inaccuracies resulting in reduced accuracy of tumor location between patient and atlas. Because glioblastomas grow in a diffusely infiltrating pattern, there is also a potential risk of different degrees of infiltration of brain structures between cases not being accounted for.

## Conclusions

Our findings suggest that there are differences in OS in glioblastoma patients based on tumor location that are not limited to eloquence. OS was short in patients with centrally located tumors and tumors in the left temporal lobe pole, and higher in patients with tumor location in the right dorsomedial temporal lobe and white matter region involving the left anterior paracentral gyrus/dorsal supplementary motor area/medial precentral gyrus. TVTB may be an important prognostic factor that can support clinical decision-making, especially in the elderly where low TVTB was associated with decreased survival, and the benefits of surgery must be carefully weighed by the surgeon.

## Supplementary Information

ESM 1 (PDF 85 kb)

ESM 2 (PDF 97 kb)

ESM 3 (PDF 97 kb)

ESM 4 (PDF 98 kb)
