# Supplementary material for: Survival of glioblastoma in relation to tumor location: a statistical tumor atlas of a population-based cohort
Source: Acta Neurochir (Wien). 2021 Mar 20;163(7):1895–905. doi: 10.1007/s00701-021-04802-6 (PMC8195961; doi:10.1007/s00701-021-04802-6)
Supplement: Supplementary file 1 — (PDF 85 kb) [file 701_2021_4802_MOESM1_ESM.pdf]

Online Resource 1

Article title: Survival of glioblastoma in relation to tumor location: A statistical tumor atlas of a population-based cohort

Journal: Acta Neurochirurgica

Even Hovig Fyllingen PhD, Lars Eirik Bø PhD, Ingerid Reinertsen PhD, Asgeir Store Jakola PhD, Lisa Millgård Sagberg PhD, Erik Magnus Berntsen PhD, Øyvind Salvesen PhD, Ole Solheim PhD

Corresponding author:
Even Hovig Fyllingen
Department of Radiology, St. Olavs Hospital, Trondheim University Hospital, Prinsesse Kristinas Gate 1, 7006 Trondheim, Norway
E-mail: even.hovig.fyllingen@gmail.com

Specifications of statistical considerations

Possible interaction terms evaluated for inclusion in the multivariable Cox proportional hazards regression model:

- age × KPS
- age × radiochemotherapy
- age × preoperative tumor volume
- age × residual tumor volume
- age × TVTB
- KPS × preoperative tumor volume
- KPS × TVTB
- radiochemotherapy × residual tumor volume
- radiochemotherapy × TVTB
- residual tumor volume × TVTB

The proportional hazard assumption for the Cox proportional hazards regression model was tested by use of scaled Schoenfeld residuals with significance level of $P \leq 0.05$. Linearity in continuous covariates was assessed visually by plotting Martingale residuals against the covariates. Any effect of influential observations was assessed by delta-beta plots with cut-off = 2/sqrt(N). Overall goodness-of-fit (GOF) was assessed by Grønnesby-Borgan test with statistical level of GOF set to $P \leq 0.1$. In the ordinal regression models age, sex, preoperative KPS, preoperative tumor volume in mL and TVTB were included in the analyses (Online resource 2). The proportional odds assumption was tested using the Brant test. For this analysis only, residual tumor volume was grouped into 0 mL to 5 mL, > 5 mL to 10 mL, > 10 mL to 15 mL, > 15 to 20 mL and > 20 mL due to heteroscedasticity, non-normality of residuals and significant outliers when analyzing residual tumor volume as a continuous variable. In the binomial regression models exploring predictors for treatment with biopsy only age, sex, preoperative KPS, preoperative tumor volume in mL and TVTB were included in the analyses (Online resource 3). In the binomial regression models exploring predictors for adjuvant radiochemotherapy age, sex,

preoperative KPS, preoperative tumor volume in mL, TVTB biopsy only and RTV were included in the analyses (Online resource 4). Linearity with respect to the logit of the dependent variable was assessed by the Box-Tidwell procedure. Multicollinearity in the logistic regression models were explored by calculation of variance inflation factor (VIF), with significant multicollinearity set to VIF > 10. Descriptive statistics were performed using IBM SPSS Statistics 26.0.0.0 64-bit on OS X 10.14, while all other analyses were performed using Stata/MP 16.0 64-bit on OSX 10.14. Regression analyses were performed by E.H.F and Ø.S, while probabilistic maps were created by I.R and L.E.B.
