# Supplementary material for: Survival of glioblastoma in relation to tumor location: a statistical tumor atlas of a population-based cohort
Source: Acta Neurochir (Wien). 2021 Mar 20;163(7):1895–905. doi: 10.1007/s00701-021-04802-6 (PMC8195961; doi:10.1007/s00701-021-04802-6)
Supplement: Supplementary file 2 — (PDF 97 kb) [file 701_2021_4802_MOESM2_ESM.pdf]

Online Resource 2

Article title: Survival of glioblastoma in relation to tumor location: A statistical tumor atlas of a population-based cohort

Journal: Acta Neurochirurgica

Even Hovig Fyllingen PhD, Lars Eirik Bø PhD, Ingerid Reinertsen PhD, Asgeir Store Jakola PhD, Lisa Millgård Sagberg PhD, Erik Magnus Berntsen PhD, Øyvind Salvesen PhD, Ole Solheim PhD

Corresponding author:
Even Hovig Fyllingen
Department of Radiology, St. Olavs Hospital, Trondheim University Hospital, Prinsesse Kristinas Gate 1, 7006 Trondheim, Norway
E-mail: even.hovig.fyllingen@gmail.com

Online Resource 2
Odds ratio (OR) of increasing residual tumor volume by patient and tumor characteristics in resected tumors (*N* = 167)

	Univariable		Multivariable	
	OR (95 % CI)[a]	*P*	OR (95 % CI)[a]	*P*
Age (per year)	1.005 (0.975-1.037)	0.744	-	-
Sex [reference female]				
Male	1.441 (0.714-2.906)	0.307	-	-
Preoperative KPS [reference < 70]				
≥ 70	0.301 (0.146-0.620)	0.001	0.439 (0.195-0.987)	0.046
Preoperative tumor volume (per mL)	1.031 (1.019-1.042)	< 0.001	1.015 (1.002-1.028)	0.022
TVTB[b] (per cm)	0.401 (0.290-0.555)	< 0.001	0.527 (0.358-0.774)	0.001

[a] Odds ratio of changing to one higher category of grouped residual tumor volumes. Residual tumor volume grouped into 0 mL to 5 mL, > 5 mL to 10 mL, > 10 mL to 15 mL, > 15 mL to 20 mL and > 20 mL.
[b] TVTB = shortest distance from center of third ventricle to preoperative contrast-enhancing tumor border.
