# Supplementary material for: Survival of glioblastoma in relation to tumor location: a statistical tumor atlas of a population-based cohort
Source: Acta Neurochir (Wien). 2021 Mar 20;163(7):1895–905. doi: 10.1007/s00701-021-04802-6 (PMC8195961; doi:10.1007/s00701-021-04802-6)
Supplement: Supplementary file 3 — (PDF 97 kb) [file 701_2021_4802_MOESM3_ESM.pdf]

Online Resource 3

Article title: Survival of glioblastoma in relation to tumor location: A statistical tumor atlas of a population-based cohort

Journal: Acta Neurochirurgica

Even Hovig Fyllingen PhD, Lars Eirik Bø PhD, Ingerid Reinertsen PhD, Asgeir Store Jakola PhD, Lisa Millgård Sagberg PhD, Erik Magnus Berntsen PhD, Øyvind Salvesen PhD, Ole Solheim PhD

Corresponding author:
Even Hovig Fyllingen
Department of Radiology, St. Olavs Hospital, Trondheim University Hospital, Prinsesse Kristinas Gate 1, 7006 Trondheim, Norway
E-mail: even.hovig.fyllingen@gmail.com

Online Resource 3
Odds ratio (OR) of biopsy only by patient and tumor characteristics (*N* = 215)

	Univariable		Multivariable	
	OR (95 % CI)	*P*	OR (95 % CI)	*P*
Age (per year)	1.138 (1.084-1.194)	< 0.001	1.149 (1.089-1.212)	< 0.001
Sex [reference female]				
Male	0.700 (0.348-1.409)	0.318	-	-
Preoperative KPS [reference < 70]				
≥ 70	0.286 (0.140-0.585)	0.001	0.630 (0.272-1.462)	0.282
Preoperative tumor volume (per mL)	1.005 (0.996-1.015)	0.287	-	-
TVTB[a] (per cm)	0.584 (0.436-0.781)	< 0.001	0.508 (0.349-0.743)	< 0.001

[a] TVTB = shortest distance from center of third ventricle to preoperative contrast-enhancing tumor border.
